# Bilateral Brachial Neuritis after COVID-19: Case Report

**DOI:** 10.1055/s-0042-1750756

**Published:** 2024-12-27

**Authors:** Yussef Ali Abdouni, Maria-Roxana Viamont-Guerra

**Affiliations:** 1 Departamento de Ortopedia e Traumatologia, Santa Casa Misericórdia de São Paulo, São Paulo, SP, Brasil; 2 Hospital Israelita Albert Einstein, São Paulo, SP, Brasil

**Keywords:** brachial plexus, Parsonage-Turner syndrome, SARS-CoV-2

## Abstract

Brachial neuritis, or Parsonage-Turner syndrome, is a rare disease characterized by a sudden, self-limiting pain in the upper limb followed by weakness and atrophy of the shoulder girdle muscles. Bilateral brachial plexus involvement occurs in between 10 and 30% of the patients, but symptoms are usually asymmetrical. The most common etiological factors include infection (25 to 55%) and autoimmune conditions. Up to 16% of the patients infected by the new coronavirus variant (SARS-CoV2) had neuromuscular complications. We present the case of a patient with bilateral Parsonage-Turner syndrome shortly after severe COVID-19.

## Introduction

Brachial neuritis, also known as Parsonage-Turner syndrome (PTS), is a rare disease with an incidence of 2 cases per 100,000 inhabitants. ^1^ Dreschefeld was the first to describe it as a shoulder girdle muscular atrophy in 2 sisters in 1887. Later, in 1948, Parsonage and Turner detailed the clinical features of the condition in a series with 136 patients. 2

Parsonage-Turner syndrome starts with sudden pain in the upper limb, with no history of trauma. It lasts from days to weeks and usually resolves spontaneously. Then, weakness and muscle atrophy begin. The neurological lesion most frequently affects the proximal portion of the brachial plexus (upper roots). This injury results in motor impairment and may cause paresthesia in the affected territory. 3 4 Bilateral involvement occurs in between 10 and 30% of the patients; however, its presentation is usually asymmetric. 5

Acute PTS can mimic cervical disc herniation, rotator cuff rupture, and adhesive capsulitis. However, the spontaneous pain improvement followed by weakness and muscle atrophy suggests PTS. 6

Although the etiology of PTS remains uncertain, triggering factors associated with immunomediated responses may play a role, including viral infections (25 to 55%), vaccination (15%), and surgical procedures (10%). 3 4 The implicated infectious agents are parvovirus B19, cytomegalovirus, Epstein-Barr virus, coxsackievirus, herpesvirus, and HIV. 6 7

In January 2020, Chinese researchers identified a novel coronavirus (SARS-CoV-2) as the etiologic agent of a severe acute respiratory syndrome called coronavirus disease 2019 (COVID-19). 8 The disease spread rapidly to the whole world and the WHO declared it a pandemic in March 2020.

Up to 16% of COVID-19 patients present neuromuscular complications. ^9^ Those who survived the severe form of the disease and underwent intensive care and mechanical ventilation may develop global weakness associated with sarcopenia and critical illness polyneuropathy. However, there are reports of other neurological conditions related to COVID-19. Needham et al. 9 described an uncommon disease, called multiple mononeuropathy, after intensive care unit (ICU) admissions. In addition to symmetrical, moderate muscle weakness resulting from sarcopenia, some patients had asymmetric deficits with no myopathy or polyneuropathy signs. Mitry et al. 10 described a patient with left shoulder paralysis after a SARS-CoV2 infection requiring no hospitalization. Articular pain started in the left shoulder a few weeks after the respiratory illness.

We present the case of a patient with asymmetric bilateral PTS shortly after SARS-CoV2 infection.

## Case Report

Male, 63-year-old, right-handed patient with no previous comorbidities started to present respiratory symptoms 11 days after a positive result at a reverse-transcriptase polymerase chain reaction (RT-PCR) for SARS-CoV2. Pulmonary involvement was > 75%, leading to admission to the ICU for 31 days, including 19 days under orotracheal intubation. The patient spent a long time in the prone position, from 16 to 24 consecutive hours, for desaturation and hypoxemia control. In total, he spent 82 hours in this position. He had mechanical ventilation-related pneumonia and received antibiotics.

The patient presented significant muscle weakness after extubation and was unable to raise the shoulders and remain in orthostasis. He started physical therapy twice a day and received protein supplements, resulting in muscle strength improvement. Discharge from the hospital occurred 14 days after the patient left the ICU, walking unaided and with no need for supplemental.

After hospital discharge, the patient underwent daily motor and respiratory physical therapy at home, with cardiopulmonary status and muscle strength improvement. However, some motor deficits persisted, including decreased active elevation of the right shoulder (< 45°) and of the left shoulder (< 20°), and a winged right scapula. The patient had normal passive range of motion and no sensory deficits.

**Fig. 1:**
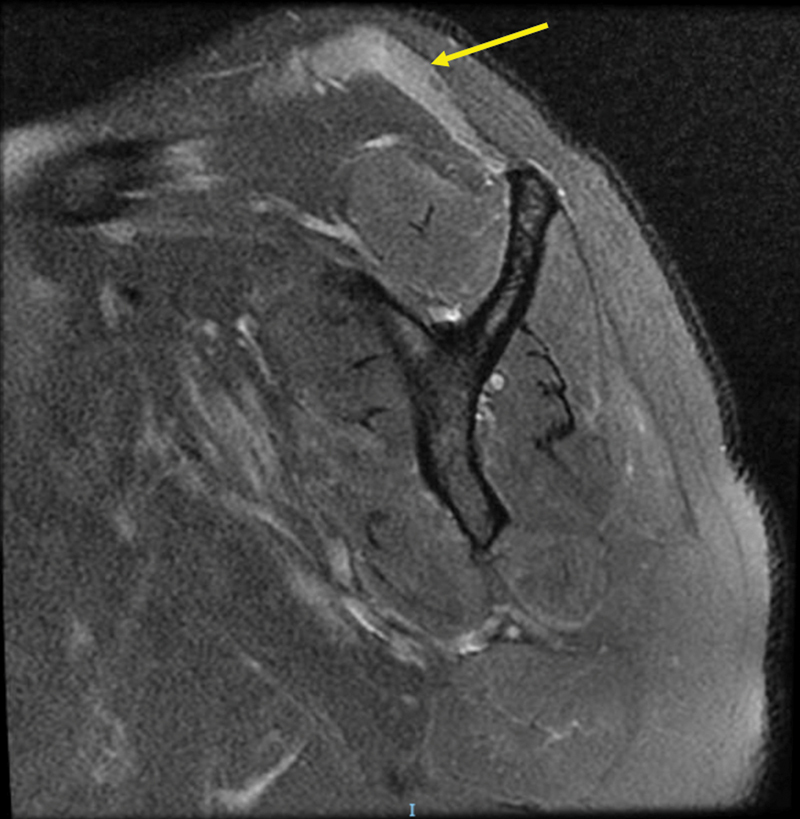
Sagittal section of the right shoulder showing a marked signal change and an edema pattern at the trapezius muscle belly (arrow).

We requested tests between the 5 ^th^ and 7 ^th^ weeks after hospital discharge. A magnetic resonance imaging (MRI) of the right shoulder revealed the following ( Fig. 1 ):

- marked signal change with an edema pattern at the trapezius muscle belly
- a mild signal change with an edema pattern at the subscapularis, teres major, and teres minor muscle bellies

**Fig. 2:**
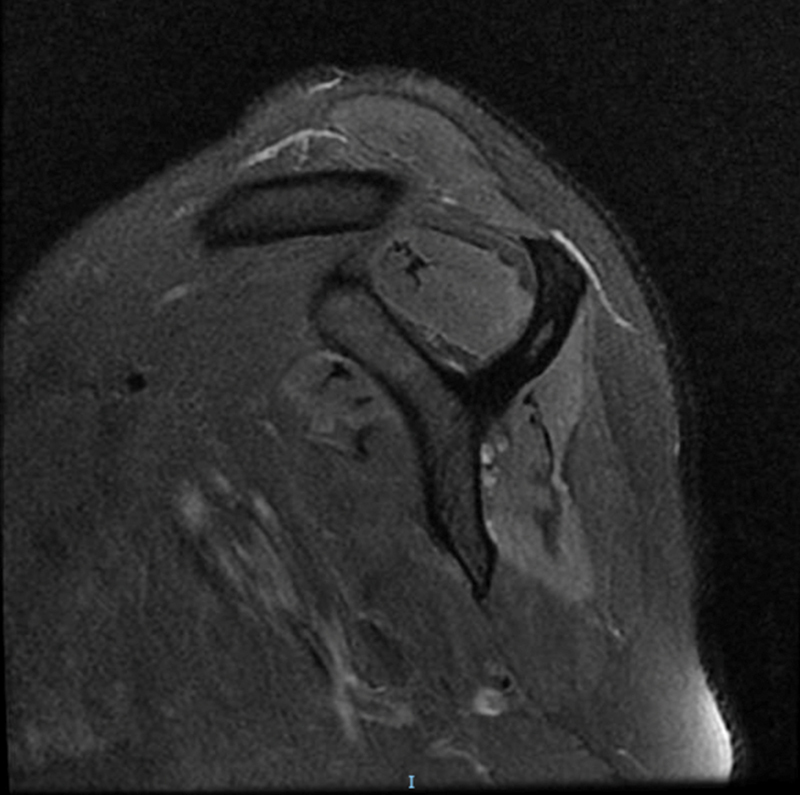
Sagittal section of the left shoulder showing a signal change and an edema pattern at the supraspinatus and infraspinatus muscle bellies. In addition, the infraspinatus muscle presents a volumetric reduction and fat replacement (Goutallier grade 1/2).

An MRI of the left shoulder revealed the following ( Fig. 2 ):

- a signal alteration with an edema pattern at the supraspinatus and infraspinatus muscle bellies, in addition to a slight volumetric reduction and fat replacement (Goutallier grade 1/2) at the infraspinatus muscle
- a mild signal change with an edema pattern at the anterior portion of the deltoid muscle belly

A cervical spine MRI showed no signs of radicular compression.

An electromyography of the upper limbs revealed the following findings:

- a marked, subacute to chronic axonal involvement of a branch of the right accessory nerve to the trapezius muscle, with signs of active denervation and no evidence of reinnervation
- moderate, subacute to chronic involvement of the myotome usually supplied by the left C5 root with evidence of recent reinnervation and signs of ongoing denervation.

## Discussion

Brachial neuritis, or Parsonage-Turner syndrome, is an uncommon condition characterized by acute pain in the upper limb. Parsonage-Turner syndrome is usually unilateral and may progress to a neurological deficit with shoulder girdle weakness and muscle atrophy. Its etiology and pathophysiology remain uncertain. Viral infections are the most common risk factor associated with PTS. ^6,7^ The literature has few reports of post-SARS-CoV2 infection PTS. ^10–12^ However, we did not detect the pain that characterizes the initial phase of the syndrome because the patient was sedated and intubated, which impaired his evaluation.

Even though the diagnosis of PTS is essentially clinical, electroneuromyography findings are abnormal and consistent in 96.3% of the patients, which may help to confirm it. ^5^ Electromyographic tests reveal acute denervation, fibrillation, and positive waves 3 to 4 weeks after the onset of symptoms. The most affected nerves include the long thoracic and suprascapular nerves. In the present case, we noticed the involvement of the accessory nerve on one side and of the C5 root, more notably the suprascapular nerve, on the other side. The accessory nerve, responsible for innervating the trapezius, is not part of the brachial plexus and is rarely affected by PTS.

Magnetic resonance imaging demonstrates signs of denervation of the affected musculature with a decreased signal on T2-weighted sequences. However, initially, other abnormalities are unlikely to be observed. Magnetic resonance imaging is still useful to rule out other causes of shoulder pain, such as rotator cuff tears, calcific tendonitis, and adhesive capsulitis. 13

Orthopedists should be aware of this potential etiology for shoulder pain. In addition, they must know how to differentiate it from the sarcopenia-related weakness that follows an intensive care period as the number of COVID-19 cases continues to increase worldwide.
