# Supplementary material for: Bilateral Brachial Neuritis after COVID-19: Case Report
Source: Rev Bras Ortop (Sao Paulo). 2024 Dec 27;59(Suppl 2):e173–5. doi: 10.1055/s-0042-1750756 (PMC11679609; doi:10.1055/s-0042-1750756)
Supplement: Related texts

# Research article: Neurite braquial bilateral pós-COVID-19: Relato de caso

**Keywords:** plexo braquial, SARS-CoV-2, síndrome de Parsonage-Turner

## Abstract

A neurite braquial, ou síndrome de Parsonage-Turner, é uma doença rara caracterizada por quadro doloroso e súbito no membro superior, autolimitado, seguido de fraqueza e atrofia da musculatura da cintura escapular. Ocorre envolvimento bilateral do plexo braquial em de 10 a 30% dos pacientes, porém os sintomas geralmente são assimétricos. Infecção viral (25 a 55%) e alterações autoimunes têm sido descritas como os fatores causais mais comuns. Uma nova variante do coronavírus (SARS-CoV2) provocou uma pandemia e complicações neuromusculares em pacientes infectados podem chegar a até 16% dos casos. Apresentamos o caso de um paciente com Síndrome de Parsonage-Turner com comprometimento bilateral logo após quadro de COVID-19 grave.

## Introdução

A neurite braquial, também conhecida como síndrome de Parsonage-Turner (SPT), é uma doença rara com incidência de 2 casos a cada 100.000 habitantes. 1 A SPT foi descrita inicialmente em 1887 por Dreschefeld, que relatou atrofia muscular da cintura escapular em 2 irmãs. Porém, foram Parsonage e Turner que, em 1948, detalharam os aspectos clínicos em uma série com 136 pacientes. 2

A síndrome inicia-se com dor súbita no membro superior, sem história de trauma, com duração de dias a semanas, geralmente com resolução espontânea. Após o quadro álgico, surge fraqueza e atrofia muscular. A lesão neurológica acomete mais frequentemente a porção proximal do plexo braquial (raízes superiores), com comprometimento principalmente motor, podendo apresentar parestesias no território da inervação afetada. 3 4 Ocorre envolvimento bilateral em de 10 a 30% dos pacientes; contudo, a apresentação da síndrome é geralmente assimétrica. 5

Na fase aguda, o quadro pode ser confundido com hérnia de disco cervical, ruptura do manguito rotador e capsulite adesiva. No entanto, a melhora espontânea da dor e a evolução com fraqueza e atrofia muscular sugerem o diagnóstico da SPT. 6

A etiologia da síndrome permanece incerta; contudo, acredita-se que existam fatores desencadeantes associados a uma resposta imunomediada: infecções virais (25 a 55%), vacinação (15%) e procedimentos cirúrgicos (10%). 3 4 Dentre os agentes infecciosos, existem relatos secundários a Parvovírus B19, Citomegalovírus, Epstein-Barr, Coxsackievirus, Herpes vírus e HIV. 6 7

Em janeiro de 2020, pesquisadores chineses identificaram um novo coronavírus (SARS-CoV-2) como agente etiológico da síndrome respiratória aguda grave denominada COVID-19 (Coronavirus Disease – 2019). 8 A doença espalhou-se rapidamente pelo mundo, sendo declarada pandemia pela OMS em março de 2020.

As complicações neuromusculares nos indivíduos com COVID-19 podem chegar a 16%. 9 É esperado que sobreviventes da forma grave da doença, submetidos a cuidados de terapia intensiva e ventilação mecânica, desenvolvam fraqueza global associada à sarcopenia e à polineuromiopatia do doente crítico. No entanto, outros quadros neurológicos relacionados à COVID-19 têm sido relatados. Needham et al. 9 descreveram os achados de uma afecção chamada mononeuropatia múltipla, quadro incomum após internações em unidades de tratamento intensivo (UTIs). Observou-se que, além da fraqueza muscular simétrica e moderada decorrente da sarcopenia, alguns pacientes evoluíram com déficits assimétricos, sem sinais de miopatia ou polineuropatia. Mitry et al. 10 descreveram um quadro de paralisia no ombro esquerdo em uma paciente que havia apresentado infecção por SARS-CoV2, sem necessidade de internação. Algumas semanas após o quadro respiratório, iniciou-se a dor articular no ombro esquerdo.

Apresentamos o caso de um paciente com SPT com comprometimento bilateral assimétrico logo após infecção por SARS-CoV2.

## Relato do Caso

Paciente do sexo masculino, 63 anos, destro, sem comorbidades prévias, apresentou sintomas respiratórios 11 dias após resultado positivo do teste de reação de transcriptase reversa seguida de reação em cadeia da polimerase (RT-PCR, na sigla em inglês) para SARS-CoV2. O acometimento pulmonar foi > 75%, levando à necessidade de internação na UTI por 31 dias, sendo 19 com intubação orotraqueal. Durante este período, o paciente ficou longos períodos em posição pronada, variando de 16 a 24 horas consecutivas, totalizando 82 horas, devido à dificuldade no controle da dessaturação e da hipoxemia. O paciente apresentou pneumonia associada à ventilação mecânica, tratada com antibioticoterapia.

Após a extubação, verificou-se importante fraqueza muscular, com incapacidade de elevar os ombros e de ficar em ortostatismo. O paciente passou a fazer fisioterapia duas vezes por dia e a receber suplemento proteico, com melhora da força muscular, recebendo alta hospitalar 14 dias após deixar a UTI, sem necessidade de suplemento de oxigênio e deambulando sem auxílio.

Após a alta hospitalar, o paciente manteve fisioterapia domiciliar motora e respiratória diariamente, com melhora da condição cardiopulmonar e da força muscular. Entretanto, observou-se persistência de alguns déficits motores: diminuição da elevação ativa do ombro direito (< 45°) e do ombro esquerdo (< 20°) e da escápula alada à direita. O paciente apresentava arco de movimento passivo normal e nenhum déficit sensitivo.

Exames complementares foram realizados entre a 5ª e a 7ª semanas após a alta hospitalar, observando-se na ressonância magnética (RM) do ombro direito ( Fig. 1 ):

- acentuada alteração de sinal com padrão de edema do ventre muscular do trapézio.
- leve alteração de sinal com padrão de edema dos ventres musculares do subescapular, do redondo maior e do redondo menor.

**Fig. 1:** Corte sagital do ombro direito mostrando acentuada alteração de sinal com padrão de edema do ventre muscular do trapézio (seta).

Observou-se na RM do ombro esquerdo ( Fig. 2 ):

- alteração de sinal com padrão de edema dos ventres musculares do supraespinhal e infraespinhal, leve redução volumétrica e lipossubstituição (Goutallier grau 1/2) do infraespinhal.
- leve alteração de sinal com padrão de edema da porção anterior do ventre muscular do deltoide.

**Fig. 2:** Corte sagital do ombro esquerdo mostrando alteração de sinal com padrão de edema dos ventres musculares do supraespinhal e infraespinhal, redução volumétrica e lipossubstituição (Goutallier grau 1/2) do infraespinhal.

A RM da coluna cervical não mostrou sinais de compressão radicular.

Verificou-se na eletroneuromiografia dos membros superiores:

- comprometimento axonal subagudo a crônico do nervo acessório direito, em seu ramo para o músculo trapézio, de grau acentuado, com sinais de denervação ativa, sem sinais de reinervação.
- comprometimento do miótomo usualmente suprido pela raiz C5 à esquerda, de intensidade moderada, subagudo a crônico, com evidências de reinervação recente e sinais de denervação em atividade.

## Discussão

A neurite braquial, ou SPT, é uma condição incomum caracterizada por dor aguda no membro superior, mais comumente unilateral, a qual pode evoluir para déficit neurológico definido por fraqueza e atrofia muscular na cintura escapular. Embora a etiologia e a fisiopatologia da doença ainda permaneçam incertas, infecção viral tem sido descrita como o fator de risco associado mais comum. 6 7 Ainda há poucos relatos de SPT após infecção por SARS-CoV2 encontrados na literatura. 10 11 12 No caso aqui descrito, no entanto, não foi observado o quadro álgico que caracteriza a fase inicial da síndrome, certamente pelo fato de o paciente estar sedado e intubado, o que prejudicou a avaliação.

Apesar do diagnóstico da SPT ser essencialmente clínico, a eletroneuromiografia é anormal e compatível em 96,3% dos pacientes, podendo ajudar a confirmar o diagnóstico. 5 Os testes eletromiográficos revelam denervação aguda, com fibrilação e ondas positivas, 3 a 4 semanas após o início dos sintomas. Os nervos mais acometidos são o torácico longo e o supraescapular. No presente caso, observou-se acometimento do nervo acessório de um lado e, do outro, da raiz de C5, mais notadamente do nervo supraescapular. O nervo acessório, responsável pela inervação do trapézio, não integra o plexo braquial e está mais raramente envolvido nesta patologia.

A RM demonstra sinais de denervação da musculatura acometida, com diminuição do sinal em T2; porém, nas fases iniciais, é provável que não sejam observadas anormalidades. A RM ainda é útil para descartar outras causas de dor no ombro, como lesão do manguito rotador, tendinite calcárea e capsulite adesiva. 13

Ortopedistas devem estar atentos a esta possível etiologia da dor no ombro, assim como diferenciá-la da fraqueza observada na sarcopenia que sucede o período de terapia intensiva, pois o número de casos de COVID-19 continua a aumentar em todo o mundo.
